# A Sweet Diagnosis for a Non-Resolving Rash

**DOI:** 10.7759/cureus.2516

**Published:** 2018-04-22

**Authors:** Jian Liang Tan, Kah Poh Loh, Kshitij Thakur, Edward F Chan, Arezoo Ghaneie

**Affiliations:** 1 Internal Medicine, Crozer-Chester Medical Center, Upland, USA; 2 Hematology and Oncology, Strong Memorial Hospital/University of Rochester Medical Center, Rochester, USA; 3 Dermatology, Hospital of the University of Pennsylvania, Philadelphia, USA; 4 Oncology, Crozer-Chester Medical Center, Upland, USA

**Keywords:** myelodysplastic syndrome, subcutaneous sweet’s syndrome, neutrophilic panniculitis, sweet’s syndrome, azacitidine-associated sweet’s syndrome

## Abstract

Subcutaneous Sweet’s syndrome (SSS) is a rare variant of Sweet’s syndrome (SS), clinically characterized by erythematous plaques or nodules with a histologic pattern demonstrating a neutrophilic panniculitis (NP). We report a case of a 74-year-old woman with myelodysplastic syndrome (MDS) who presented with persistent fever, malaise, and non-resolving generalized erythematous nodules and was found to have an MDS-related SSS. SSS should be entertained and other causes of NP should be excluded prior to treating a patient with systemic corticosteroids. Early diagnosis of SSS in a patient not responding to broad-spectrum antibiotics is crucial as it helps to minimize unnecessary prolonged antibiotics exposure in this era of antimicrobial resistance. In patients with frequent relapses, a slow corticosteroid taper could be beneficial.

## Introduction

Sweet’s syndrome (SS), also known as acute febrile neutrophilic dermatosis, was first described in 1964 [1]. Subcutaneous Sweet’s syndrome (SSS) is a rare variant of Sweet’s syndrome (SS), clinically characterized by erythematous plaques or nodules with a histologic pattern demonstrating a neutrophilic panniculitis (NP) [2-3]. Primary NP is characterized by neutrophilic infiltrates exclusively in the subcutaneous fat with minimal dermal involvement [2-3].

The diagnostic criteria of SS and SSS differ by their respective subtle histopathological descriptions, where the main neutrophilic infiltration site involvements are the dermis versus the subcutaneous tissue. The diagnosis of SSS should be suspected based on the relevant clinical and histologic findings (especially in the setting of myeloid disorders with primary NP) after excluding other important causes of NP, including infectious panniculitis, early erythema nodosum, and leukemia cutis [4-5]. We hereby present a case of myelodysplastic syndrome (MDS)-related SSS that responded to oral prednisone.

## Case presentation

Informed consent was obtained from the patient prior to the submission of this paper.

**Figure 1:**
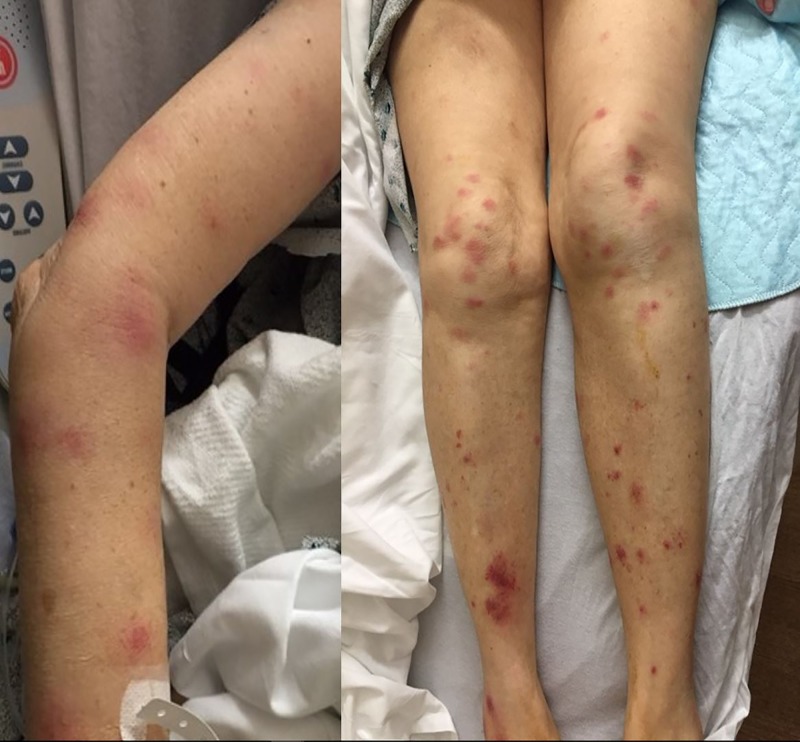
Tender erythematous plaques and nodules on the lower extremities and forearm

A 74-year-old Caucasian woman presented with fever, fatigue, and painful erythematous nodules. Her oncologic history was significant for MDS (refractory cytopenia with multilineage dysplasia subtype) diagnosed three years previously. She received 23 cycles of azacitidine (AZA). On initial presentation, her temperature was 101.4° F with tachycardia. Physical examination was significant for conjunctival pallor, tender erythematous vesicles on her right temple and bilateral ear lobes extending to the right periocular area, and tender erythematous nodules on her buttocks. A complete blood count showed pancytopenia (white blood cell count of 2.0 x 10­^9^/L with an absolute neutrophil count of 1,500/mm^3^, hemoglobin of 8.1 g/dL, and platelet count of 16 x 10^9^/L). Given the concern for sepsis, she was started on antibiotics (1 gm of vancomycin and aztreonam every 12 hours) and antiviral medications (650 mg of acyclovir every eight hours). Despite that, she was persistently febrile with worsening of her condition and development of new erythematous plaques and nodules over her shoulders, forearms, and lower extremities (Figure *1*).

**Figure 2:**
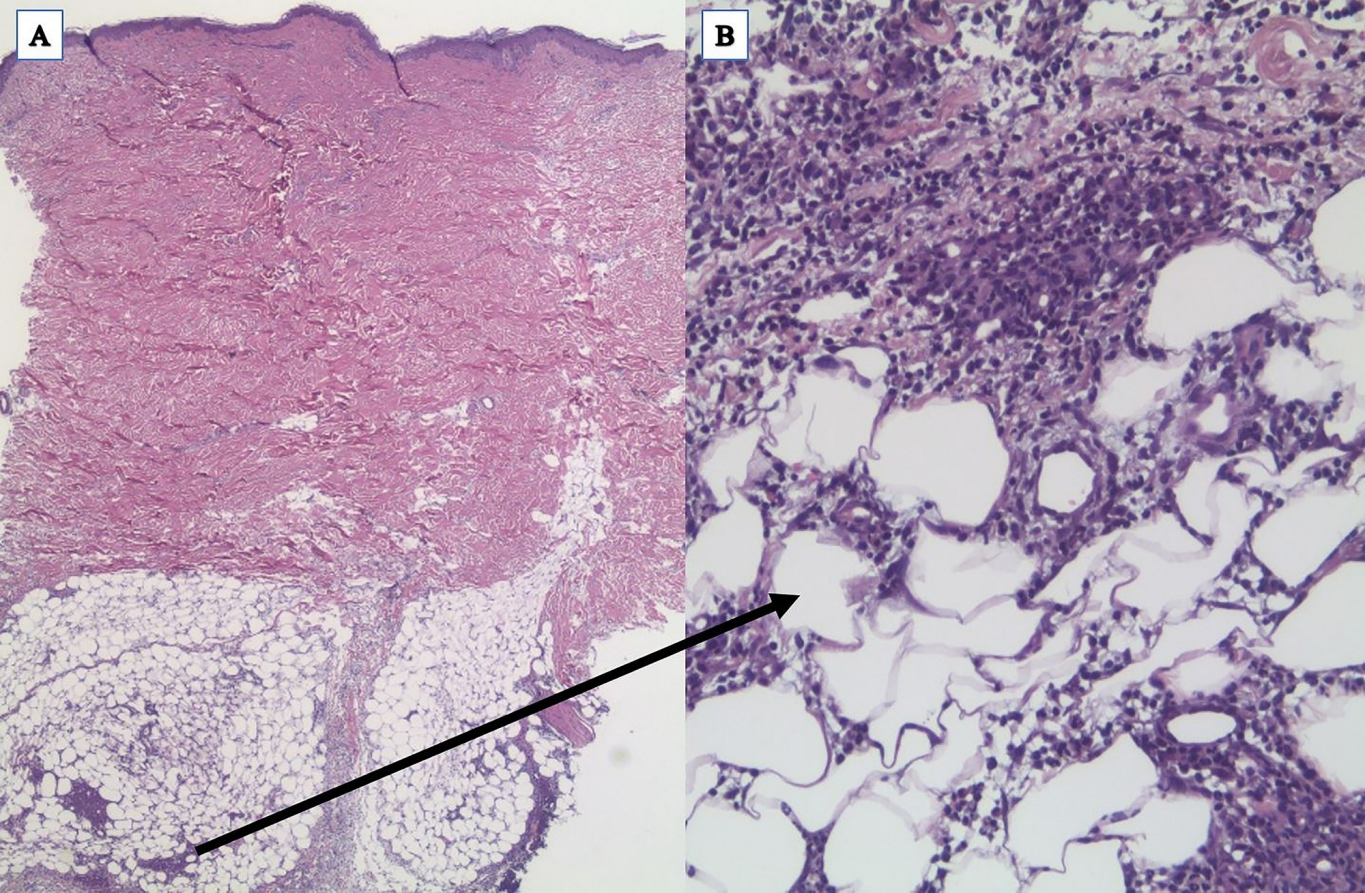
Histopathology of the skin biopsy A 5-mm x 5-mm x 6-mm punch biopsy was performed. It revealed nodular aggregates of mature neutrophils and scattered histiocytes within the subcutaneous adipose tissue with no involvement of the dermis. No evidence of vasculitis or myeloid was noted. These histomorphological features were consistent with neutrophilic panniculitis. (Hematoxylin-eosin stain; original magnifications: A, x25; B, x200)

A 5-mm x 5-mm x 6-mm punch biopsy of an erythematous nodule over her right shoulder showed subcutaneous lobular and septal infiltrates of neutrophils and scattered histiocytes with sparing of the dermis, consistent with NP (Figure *2*). 

Special stainings (Gram's method, Ziehl-Neelsen, and Periodic acid–Schiff stains) and tissue cultures for bacteria, mycobacteria, and fungal infections were negative. Blood cultures were negative for infection. Bone marrow biopsy did not show evidence of transformation of her MDS to acute myeloid leukemia (AML). A diagnosis of MDS-related SSS was made. She was started on oral prednisone, 60 mg/day, and had marked clinical improvement with resolution of her fever within 24 hours. Although she had relapsed painful erythematous nodules and plaques with a rapid steroid taper, she reported resolution of her cutaneous lesions weeks later at follow-up with a slow steroid taper. In the meantime, AZA was withheld.

## Discussion

SS, first described in 1964, is classified into three subgroups: classic or idiopathic SS, malignancy-related SS, and drug-induced SS. Malignancy-related SS accounted for around 21% of cases and most of them were due to hematologic malignancy (85%) [1, 6]. AML itself made up 42% of all the hematologic malignancies, whereas MDS accounted for only about 22% [7-8]. Von den Driesch proposed two major criteria and four minor criteria to define SS. The diagnosis of SS requires both of the two major criteria and two of the four minor criteria (Table 1) [9]. 

**Table 1:** Diagnostic Criteria for Classical and Malignancy-related Sweet's Syndrome ESR: erythrocyte sedimentation rate; WBCs: white blood cells

Major Criteria	Sudden onset of painful erythematous nodules Dense neutrophilic infiltrates in the dermis without leukocytoclastic vasculitis
Minor Criteria	General malaise and fever > 38° C Association with underlying hematologic malignancy or solid tumors, inflammatory diseases, or pregnancy, or preceded by vaccination, gastrointestinal, or upper respiratory tract infection Three of four of these abnormal laboratory values upon presentation: ESR > 20 mm/hr; C-reactive protein positive; WBCs > 8,000; > 70% neutrophils Excellent response to treatment with systemic steroid or potassium iodide

MDS are clonal hematopoietic stem cell disorders, characterized by hematopoiesis failure, with an increased risk of evolving into AML. A wide range of cutaneous lesions has been associated with MDS, such as SS, pyoderma gangrenosum, and erythema nodosum [4-5]. SS may precede the diagnosis of MDS or even signify the progression of underlying MDS to AML [2, 6]. As in our case, a bone marrow biopsy was performed and showed no evidence of AML transformation.

Our patient fulfilled the diagnostic criteria of SS with the exception that the neutrophil infiltration is within the subcutaneous tissue and not the dermis. Guhl et al. proposed that SSS is a rare variant of SS, clinically characterized by erythematous plaques or nodules, which has a histopathologic pattern of primary neutrophilic panniculitis (NP) [3]. Primary NP is characterized by neutrophilic infiltrates exclusively in the subcutaneous fat with minimal or no dermal involvement as seen in Figure 2 [3]. Hence, the diagnosis of SSS was made. Other possible causes of NP include infectious panniculitis, panniculitic id reaction, early erythema nodosum, and leukemia cutis.

In a patient with a myeloid disorder, it is crucial to exclude infection as a cause of NP (such as infectious panniculitis and panniculitic id reaction) prior to starting an immunocompromised patient with systemic corticosteroid treatment. A careful histologic examination of the skin biopsy often reveals basophilic necrosis, discrete abscesses, granulomas, neutrophil-lined microcysts, and vasculitis in infectious panniculitis [10]. Special staining for bacteria, mycobacteria, and fungi should be performed in addition to tissue culture to safely rule out infectious panniculitis. As in our case, none of those findings were positive to support the diagnosis of infectious panniculitis.

Panniculitic id reaction represents an abnormal immune reaction secondary to disseminated infectious antigens. A detailed history taking would reveal a history of concomitant distant infection. Useful histologic clues include leukocytoclastic vasculitis, granulomatous vasculitis, or thrombotic microangiopathy [11]. The lack of these findings in our patient, therefore, made this diagnosis highly unlikely.

Erythema nodosum should be considered as part of the histologic differential diagnosis in a patient with NP. It is frequently difficult to distinguish erythema nodosum from SSS. It was thought that the presence of Miescher's radial granuloma in the skin biopsy and a response to a trial of nonsteroidal anti-inflammatory drugs (NSAIDs) would support the diagnosis of erythema nodosum [12]. Although our patient was not given a trial of NSAIDs, the lack of Miescher's radial granuloma in the skin biopsy argued against the diagnosis of erythema nodosum.

Leukemia cutis can be a cutaneous manifestation of leukemia. The diagnosis of leukemia cutis could be made if there is a presence of myeloid blasts, which could be identified via histologic examination with CD33 and CD 117 immunostains [13]. These immunostains were not performed in our patient during the histologic examination as our patient did not bear the diagnosis of leukemia, and based on the bone marrow biopsy, there was no evidence of AML transformation. Hence, lacking such criteria would militate against the diagnosis of leukemia cutis.

Recently, there have been reports on AZA-associated SS [14]. To diagnose drug-related SS, all the five criteria would have to be met (Table *2*).

**Table 2:** Diagnostic Criteria for Drug-related Sweet's Syndrome

Sudden onset of painful erythematous nodules Dense neutrophilic infiltrates in the dermis without leukocytoclastic vasculitis General malaise and fever > 38°C Temporal relationship between medication administration and clinical manifestation or recurrence of symptoms post-medication challenge Temporally-related resolution of skin lesions after stopping the medication or treatment with systemic steroid

Notably, our patient had been on 23 cycles of AZA since 2014 prior to developing SSS in 2016. The average time to the onset of clinical symptoms in drug-induced SS was reported to be around 7.5 days [15]. In our patient, although there was a temporal relationship between medication administration and resolution of the skin lesions, the association between AZA and SSS remains unclear.

The treatment for both SSS and SS consists of systemic corticosteroids. In some of the reported cases, relapses or recurrence of cutaneous lesions occurred upon rapid steroid taper (as in our case) [1-2]. Hence, a slow steroid taper can potentially be helpful in such cases. Rarely, SSS may resolve spontaneously without any treatment, as reported by Chan et al. [2]. Notably, Agarwal et al. successfully treated a drug-refractory SSS case (failed slow steroid tapering and dapsone treatment) with adalimumab [16]. Essentially, treatment directed towards the underlying myeloid disorder or the discontinuation of the culprit drug in the case of drug-induced SSS might be the ultimate cure in a truly refractory SSS.

## Conclusions

We report a case of a patient with a history of MDS presenting with fever and erythematous rashes not responding to multiple antimicrobials who was eventually diagnosed with SSS. The diagnosis of SSS was based on meeting the criteria for SS with histopathological findings of NP and ruling out other causes of NP. A meticulous integration of all relevant clinical data and laboratory results are important in the early recognition and treatment of SSS as this could potentially avoid unnecessary prolonged exposure of a patient to the board-spectrum antibiotics. Other possibilities, including early erythema nodosum, infectious panniculitis, panniculitic id reaction, and leukemia cutis, should be excluded prior to treating the SSS with systemic corticosteroids.
